# Decreased expression of C-erbB-2 and CXCR4 in breast cancer after primary chemotherapy

**DOI:** 10.1186/1479-5876-10-S1-S3

**Published:** 2012-09-19

**Authors:** Shi-Xin Yang, Wings TY Loo, Louis WC Chow, Xin-hua Yang, Yi Zhan, Lin-Jun Fan, Fan Zhang, Li Chen, Qing-liang Wang, Hua-Liang Xiao, Jin-Long Wu, Xiu-wu Bian, Jun Jiang

**Affiliations:** 1 The Third Hospital of Nanchang, Jiangxi, PRC; 2 UNIMED Medical Institute and Organisation for Oncology and Translational Research, Hong Kong SAR; 3 Breast Disease Center, Southwest Hospital, Third Military Medical University, Chongqing, PRC; 4 Institute of Pathology, Southwest Hospital, Third Military Medical University, Chongqing, PRC

## Abstract

**Background:**

Biological molecular markers such as proto-oncogene erbB-2 (*HER-2*/*neu*, *c-erbB-2*), the CXC chemokine receptor 4 (CXCR4), estrogen receptor (ER), Proliferating Cell Nuclear Antigen (PCNA), DNA topoisomerase II (topo II), P-glycoprotein (P-gp) and glutathione S-transferase (GST) were observed for changes after administration of neochemotherapy and whether these protein expression changes were correlated with response to chemotherapy.

**Methods:**

Sixty-four patients with primary breast cancer who had undergone neo-adjuvant chemotherapy were enrolled in the present study. The expressions of C-erbB-2, CXCR4 and ER-α were measured by immunohistochemistry (IHC) on full tissue sections and on tissue microarrays (TMAs). PCNA, TopoII, P-gp and GST were measured by IHC on TMAs. On the other hand, CXCR4, C-erbB-2 and ER-α expressions were detected using western blot analysis to 16 pairs of fresh preoperative core biopsies. The final surgical specimens were obtained from patients with breast carcinoma who received neo-adjuvant chemotherapy and obtained a partial response (PR).

**Results:**

Our data demonstrated that the levels of C-erbB-2, CXCR4 and ER-α in patients decreased after they received neo-adjuvant chemotherapy on full tissue sections and on TMAs. The PCNA level was down-regulated after receiving neo-adjuvant chemotherapy, and no significant change was observed for TopoII, P-gp and GST. The levels of C-erbB-2, CXCR4 and ER-α were also down-regulated after neo-adjuvant chemotherapy was administered, as detected by western blot. In addition, the change expressions of C-erbB-2 and CXCR4 in specimens tended to be correlated with pathological change to neo-adjuvant chemotherapy on full tissue sections and on TMAs in a Pearson chi-square analysis.

**Conclusions:**

As demonstrated in our study, after breast cancer patients were treated with neo-adjuvant systemic therapy, decreased expressions of C-erbB2, ER-α and CXCR4 were observed. Down-regulated expressions of c-erbB-2 and CXCR4 may be a novel mechanism of chemotherapy; the changes of these objective markers may be useful in evaluating the clinical response of neo-adjuvant chemotherapy in breast cancer.

## Background

Chemotherapy is widely administered in the primary treatment of breast cancer. However, a subset of patients undergoing neo-adjuvant therapy suffers from side effects without benefiting from treatment. Furthermore, in patients with progressive disease, valuable time for efficient treatment is lost. The only chance to improve survival for breast cancer patients is to select timely, aggressive, and optimal therapy at the time of diagnosis. Any improvement in selecting patients who have a better than average chance to benefit from a given chemotherapy regimen is an important improvement over the current unselected empirical use of various adjuvant chemotherapy regimens. Given these facts, finding a method to evaluate the response to neo-adjuvant chemotherapy would be important. Molecular change may more accurately evaluate the effects of chemotherapy than a method based on morphological change alone. This study assesses whether some important biological molecular markers such as C-erbB-2, CXCR4, PCNA, TopoII, P-gp and GST changed after administration of neo-adjuvant chemotherapy and whether these protein expressions or change expressions were correlated with neo-adjuvant chemotherapy.

## Methods

### Patients and clinical specimens

Sixty-four breast cancer patients who received neo-adjuvant chemotherapy and underwent surgery between January 2004 and September 2006 at the Breast Disease Center, Southwest Hospital of Third Military Medical University, Chongqing were included in this study. Forty-eight pairs of core biopsies and final surgical breast cancer specimens were formalin-fixed and paraffin-embedded. The remaining 16 pairs of fresh preoperative core biopsies and final surgical specimens were stored at a temperature of -80°C.

### Treatment

Patients received CEF treatment (5-fluorouracil; epirubicin; cyclophosphamide) or ET treatment (epirubicin and Taxol) for one to six cycles (I-II stage for one- two cycles; III-IV stage for three-six cycles). No patient received tamoxifen as part of her neo-adjuvant treatment. Patients were scheduled to undergo surgery after preoperative chemotherapy with tumor excision and axillary node dissection.

### Tumor tissue microarrays (TMAs) construction

One TMA was constructed with 0.6 mm-diameter, single punches from formalin-fixed, paraffin-embedded core biopsies and surgical resection specimens of 48 patients with breast cancer, using a tissue-arraying instrument (Beecher Instruments®, Silver Spring, MD, USA). For each tumor, two representative tumor areas were carefully selected from a hematoxylin- and eosin-stained section of a donor block. Core cylinders were punched from each of these areas and deposited into a recipient paraffin block. Consecutive 4 μm-thick TMA sections were cut and placed on charged Poly-L-Lysine-coated slides for immunohistochemistry analyses.

### Immunohistochemical staining (IHC) of biomarkers

Immunohistochemistry analyses were performed using DAKO the EnVision^TM^ IHC Detection System Kit as described by Yang et al [1]. The primary antibodies used were Monoclonal Mouse Anti-Human c-erbB2 (ZYMED Co., USA), Monoclonal Mouse Anti-Human CXCR4 (Sigma), Monoclonal Mouse Anti-Human Estrogen Receptor α (Gene Tech), Monoclonal Mouse Anti-Human PCNA (Gene Tech), Mouse Anti-HumanTopoII a (Gene Tech), Mouse Anti-Human P-gp (Gene Tech) and GST (Gene Tech).

### Evaluation of immunohistochemical staining

C-erbB-2 was localized in the cell membrane and was scored semi-quantitatively using the following Food and Drug Administration (FDA)-approved scoring system [2]. For immunohistochemical staining of CXCR4, a combination of membrane and cytoplasm staining was observed in samples. The immunostaining score of CXCR4 was calculated by the multiplication of the percentage of positive tumor cells (0–100) by the staining intensity (grade 1–4), producing a total range of 0–400 [3,4]. Standard clinical factors such as ER-a, PCNA, TopoII, P-gp and GST have been evaluated routine methods in our test.

### Western blot analysis

Approximately 40 µg of total protein was boiled in a loading buffer for 5 minutes, subjected to electrophoresis through a 4%–12% polyacrylamide gradient Tris-glycine gel, along with Biotinylated Protein Ladder Detection Pack (Cell Signaling Technology, Inc.), and electroblotted onto a PVDF membrane (Dupont, USA). The Western Blotting Chemiluminescence Regent Plus Kit (Mouse/Rabbit) (ShangHai ShengNeng BoCai Biotechnology Co., Ltd.) was used for the detection of proteins. The membrane was briefly incubated for 1 hour at room temperature in 1% blocking solution and for 1 hour at 4 °C in a dilution of 1:500 Rabbit anti-Neu antibody (185Kd, Santa Cruz Biotechnology, Inc.) or ERα (66kDa, Santa Cruz Biotechnology, Inc.) or Rabbit anti-CXCR4 (43Kd, Affinity BioReagents, ABR) and 1:1000 Mouse monoclonal Beta-actin Antibody (42Kd, Novus Biologicals) in 0.5% blocking solution. The membrane was washed two times in PBS, and then with Biotin-labeled secondary antibody in a dilution of 1:2500 (Sigma, USA), incubated for 60 minutes at 4 °C, and then anti-Biotin HRP antibody is applied and detected with avidin conjugated to horseradish peroxidase (DAKO, USA). All blots were scanned with the Image Quant software using an electrochemifluorescence (ECF) Western blotting detection system (Labworks^TM^ Analysis Software, USA).

### Assessment response by pathological change and physical examination

Pathologic response was classified according to Abrial et al [5]. The clinical baseline and preoperative measurements were obtained with a caliper by the same medical oncologist. Clinical responses were recorded according to the criteria of International Union Against Cancer (UICC). Pathologic response class 1-2 is equivalent to CR and class 3, 4 is equal to PR and SD, respectively.

### Statistical analyses

The tests of statistical significance in this study were determined by Pearson’s chi-square (*χ*^2^) test and by Wilcoxon signed-rank test. In all tests, *P* < 0.05 was considered as statistically significant. All tests of statistical analyses were performed using SPSS Statistical Software Version 18.0.

## Results

### Clinical outcome

Sixty-four patients who received primary chemotherapy were included in the study. The tumor samples included (56 of 64) 87.5% invasive ductal, (8 of 64) 12.5% invasive lobular. The grade distribution was 24% grade 1, 40% grade 2, and 36% grade 3. The average age of a patient was 47 years (range: 22–71); 51.56% (33of 64) of the patients had lymph node-negative disease, while 48.44% (31of 64) had lymph node-positive disease. After the administration of a median of two courses of chemotherapy (range: 1-4), the pathological response change class 1-3 (equal to clinical CR and PR) [5] to primary chemotherapy was 41.7% (20 of 48 patients). The pathological response class 1, 2, and 3 was obtained in 4.2, 6.3, and 27.1% of the cases (1, 3, 15 of 48 patients, respectively). No PD under treatment was observed. The median number of excised axillary lymph nodes was 17.

### Immunohistochemistry

**Figure 1:**
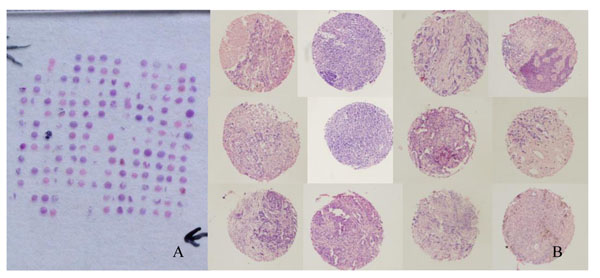
**H&E staining of a paraffin block section and enlarged image of tissue sample from tissue microarray** A: H&E staining of a paraffin block section (25 × 25 mm) from the TMA containing 192 arrayed samples, including 48 pairs of preoperative core biopsies and final surgical primary breast cancer specimens. B: The enlarged image of representative tissue sample from a tissue microarray (10×10).

**Figure 2:**
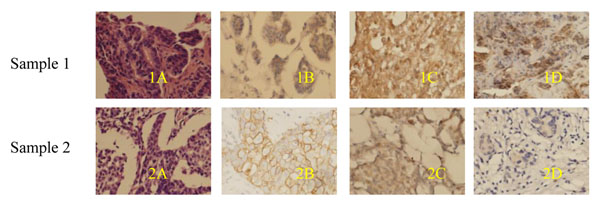
**C-erbB-2, CXCR4 and ER-α expressions in human breast tumor tissues pre- and post-treatment sample** Sample 1: Core biopsy before treatment. 1A: H&E staining; 1B: C-erbB-2 positive staining; 1C: CXCR4 positive staining; 1D: ER-α positive staining. Sample 2: Excised specimen following chemotherapy. 2A: H&E staining; 2B: C-erbB-2 weakly positive staining after chemotherapy; 2C: CXCR4 weakly positive staining after chemotherapy; 2D: ER-α negative staining after chemotherapy. (Original magnification, 400×)

The expression level of C-erbB-2 and the change was compared between pre- and post-therapy samples by IHC on TMAs (Figure 1) and full tissue sections (Figure 2, 1B and 2B). C-erbB-2 was found to be positive before and after chemotherapy in 34.8%, 37.5% and 23.8%, 9.3% of the patients, respectively. The results were determined to be statistically significant (x^2^: 10.273, 13.201; p:0.00, 0.00, respectively), as shown in Table 1. For the CXCR4 expression (Figure 2, 1C and 2C), 28 cases (72.51%) showed positive staining with down-regulated expression in 22 (56.4%) cases and up-regulated expression in 3 (7.6%) cases, whereas 12 cases (28.2%) did not display any change on full tissue sections. Overall, the expression levels of CXCR4 were compared between pre- and post-therapy samples by IHC on full tissue sections, where they were found to be statistically significant (Z: -4.168, 3.331, *p*: 0.015, 0.00 by Wilcoxon signed-rank test). Correlation was found for the change expressions of C-erbB-2 and CXCR4 on the two methods (r= 0.693, 0.457, *p*: 0.00, 0.001, respectively, by Pearson chi-Squre). For the ER-α expression, 18 cases (37.5%) showed positive cytoplasm staining with down-regulated expression in 12 (25%) cases and up-regulated expression in 3 (7.6%) cases, whereas 27 cases (56.25%) did not display change on full tissue sections (Figure 2, 1D and 2D). The expression levels of ER-α were compared between pre- and post-therapy samples by IHC on full tissue sections. They were found to be statistically significant (x^2^: 8.39; *p*: 0.040). After primary chemotherapy, the percentage of cells expressing PCNA was significantly reduced (pre-treatment versus post-treatment median value: 43.63% versus 27.89%; *p* < 0.001 by Wilcoxon signed-rank test). Interestingly, on a macroscopic level, not only C-erbB-2, CXCR4 and ER-α positive stain cells were eliminated, but membrane and cytoplasma stains were also reduced after chemotherapy, as we observed. There were no significant changes in the expressions of TopoII, P-gp and GST before and after chemotherapy.

**Table 1:** Expression status of C-erbB-2 in pre-chemotherapy and post-chemotherapy specimens on the full tissue sections.

C-erbB-2 expression	Pre-chemotherapy (%)	Post-chemotherapy (%)	χ^2^	*p*
–	13	18	---	---
+	17	14	10.273	0.016
+ +	6	8	---	---
+ + +	10	2	---	---

### The analysis of expression levels of C-erbB-2, CXCR4, ER-α between pre- and post-therapy samples with western blots

**Figure 3:**
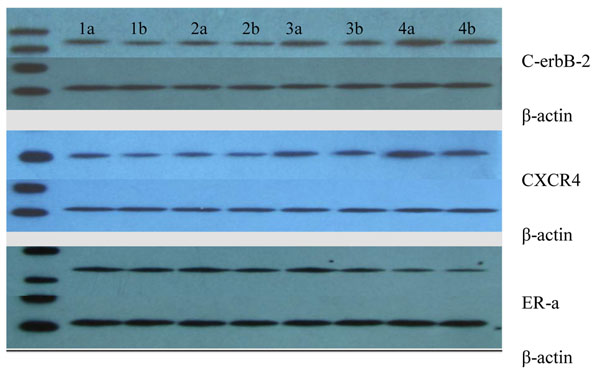
**Western blot analysis: Expressions of C-erbB-2, CXCR4 and ER-a in breast cancer** Fifty micrograms of total protein of tissue lysates from various breast cancer tissue were resolved on 4–15% SDS–PAGE and subjected to western blot analysis using antibodies that recognize phospho-specific C-erbB-2, CXCR4 and ER-a respectively. 1a-4b: 4 pairs of preoperative core biopsies and final surgical specimens. a: preoperative specimens; b: postoperative specimens.

The expression levels of C-erbB-2 were compared between pre- and post-chemotherapy samples, western blot results are shown in Figure 3. Table 2 details the expression levels of C-erbB-2 that were found to be down-regulated after administration of chemotherapy (Z: -4.168, *p*: 0.012).

**Table 2:** Analyses of expression levels of C-erbB-2, CXCR4, ER-a/*β*-actin (IOD) between pre- and post-therapy samples with western blotting

Parameters	Pre-chemotherapy specimens	Post-chemotherapy specimens	Z	*p*
C-erbB-2	0.546±0.182	0.442±0.142	-3.103	0.002
CXCR4	0.908±0.341	0.829±0.277	-2.689	0.002
ER-α	0.701±0.174	0.559±0.126	-3.516	0.00

* *p*<0.05, compared with post-chemotherapy specimens

### The changes of C-erbB-2, CXCR4, and ER-α expressions with pathological response

**Figure 4:**
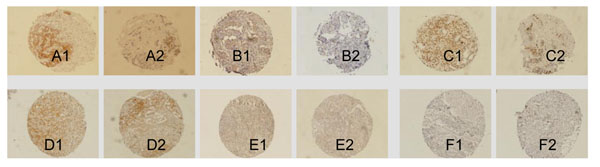
A: Anti-C-erbB-2 staining. B: Anti-CXCR4 staining. C: Anti-PCNA staining. D: Anti- GST staining. E: Anti- P-gp F: Anti-ToP staining. 1: Pre-chemotherapy core biopsy breast cancer specimens; 2: Post-chemotherapy (at the time of surgery) surgical breast cancer specimens. (Evision^+TM^ System×100).

Analyzed by IHC on full tissue sections and on TMAs (Figure 4), the change expression of C-erbB-2 in specimens tended to be correlated with pathological response to neo-adjuvant chemotherapy (χ^2^: 9.697 and 7.141, respectively; *p*: 0.046 and 0.028, respectively). The change expression in the response cases (pathological response for stages I-III) was significantly higher than the no response cases (pathological response for stage IV). Decreased expressions of C-erbB-2 and CXCR4 in specimens tended to be correlated with pathological response to neo-adjuvant chemotherapy (r: -0.320, -0.312; *p*: 0.027, 0.037, respectively) (Table 3)**.** No correlation was observed for change expressions of ER-α in specimens with pathological response to neo-adjuvant chemotherapy and on TMAs (r: -0.299; *p*: 0.073). No correlation was observed between pretreatment C-erbB-2, CXCR4, ER-α, PCNA, TopoII, P-gp and GST status with pathological response to neo-adjuvant chemotherapy on TMAs (r: -0.253, -0.008, -0.23, -0.029, -0.254, 0.182 and -0.140, respectively; *p*: 0.106, 0.972, 0.149, 0.909, 0.362, 0.571 and 0.605).

**Table 3:** Relation between the change of CXCR4 expression with pathological response in tumor tissue sample from 48 cases of primary breast cancer

		CXCR4 staining				
Pathological change	Increased	Decreased	Unchanged	χ^2^	*R*	*p*
Class 1-3	1	2	9			
Class 4	2	21	10	8.03	-0.312	0.037

Pathologic response class 1-3 equals to Clinical responses (CR) and Pathologic response (PR), class 4 equals to SD.

## Discussion

Several studies have addressed the theory that gene expression patterns might be able to predict the response of a primary breast tumor to neo-adjuvant chemotherapy**,** but according to Juliane *et al.*, [6] neo-adjuvant chemotherapy results in alterations in gene expressions, and gene expression patterns cannot predict responses to neo-adjuvant chemotherapy. Several studies have addressed the hypothesis that the changes in the expression of some biological markers, such as proliferation indices, proliferating cell nuclear antigen, expression of P-gp, and epidermal growth factor receptor and CD34, might be a response to primary chemotherapy in breast cancer [7-12]. Recently, there were also reports that stated neo-adjuvant chemotherapy also resulted in alterations in molecular markers such as estrogen and progesterone receptor (ER/PR) protein expressions [13]. In this study, we attempted to identify whether some important biological molecular markers such as C-erbB-2, CXCR4, PCNA, TopoII, P-gp and GST changed after patients received neo-chemotherapy and whether the change in protein expression was correlated with neo-adjuvant chemotherapy.

IHC on TMA offers a powerful tool to quickly evaluate the clinical relevance of differentially expressed genes and proteins [14]. TMAs may simultaneously test a large series of tumor samples and test for the same condition. Our study demonstrates that C-erbB-2 and CXCR4 protein expressions in breast cancer detected by TMAs were high concordance with that of the full sections, demonstrating that reliable results could be obtainable in detecting expressions of protein by TMAs. Evaluated by TMA, there were no significant changes observed in the expressions of TopoII, P-gp and GST after chemotherapy.

It was shown in this study that primary system chemotherapy resulted in a significant decrease in the expressions of C-erbB-2, CXCR4 and ER-α. Quddus [15] also found HER-2/neu expression decreased after neo-adjuvant chemotherapy was administered in patients with locally advanced breast cancer. However, contradictory findings have also been reported; for instance, in Tinari’s experimental study [16], no significant change was observed for the expression C-erbB-2 after neo-adjuvant chemotherapy. It should be emphasized that a lack of standardization of IHC assay renders different results. For example, Tinari [16] regarded 0 and 1+ as negative. A large portion of the changes in C-erbB-2 expression from stain + to - might have been neglected when conducting the research. Weak HER-2 expressions (score 1+) was observed in approximately 17 of 48 cases (35.4%) in our study. Weak HER-2 expressions should be considered when comparing expressions for pre- and post-chemotherapy. We observed a change of HER-2/neu status following treatment: after patients were treated with chemotherapy, the C-erbB-2 positive stained cells were eliminated, and membrane stains were also reduced (Figure 2), from positive to negative and from scores of 1+ to scores of 0.

IHC is a useful technique for visualizing proteins and for localization, but its extremely difficult techniques for quantifying results and comparing values—such as intra-tumor heterogeneity, differences in specimen processing, and technical variables—may contribute to the expression changes of molecular protein statuses [15]. Western blot, also a widely used analytical technique, contains an internal standard that allows for comparison between samples and quantification of protein expressions. Western blot may be a reasonable means to overcome technical variables such as intra-tumor heterogeneity. We detected C-erbB-2, CXCR4, ER-α expressions using western blot analysis and found that their levels down-regulated after neo-adjuvant chemotherapy.

We analyzed the relationship between changes in the expressions C-erbB-2, CXCR4 and ER-α with their pathological change after chemotherapy. Our results show that decreased expressions of C-erbB-2 and CXCR4 tended to be correlated with pathological response to neo-adjuvant chemotherapy. There was no correlation observed for **c**hanges in the expression of ER-α in specimens with pathological response to neo-adjuvant chemotherapy. Overexpression of c-erbB-2 and CXCR4 in breast cancer was considered a risk factor and was associated with the shortest survival times. Our results showed that good responders exhibited more down-regulation in c-erbB2 and CXCR4 expressions after receiving chemotherapy than did the poor responders. These results raise the possibility that neo-adjuvant chemotherapy may have eliminated c-erbB2 and CXCR4 positive tumor cells or/and reduced membrane and cytoplasmic stains. This may be a possible explanation for chemo-resistance following an initial period of chemosensitivity [15].

After treatment of neo-adjuvant systemic therapy in decreased expressions of C-erbB2 and CXCR4 in breast cancer, the findings are also biologically plausible since these functional proteins (i.e., apoptosis, invasion, metastasis, drug resistance/metabolism, proliferation) may represent effect, sensitivity and resistance to chemotherapy. Such information may be useful in evaluating the effectiveness of therapy. It may help us to better understand the molecular mechanisms of cancer metastasis and the mechanism of chemotherapy in decreased expressions of CXCR4 and C-erbB-2 which may be correlated with chemotherapy. This result has an interesting clinical implication, namely patients with decreased expressions of CXCR4 and C-erbB-2 after administration of neoadjuvant chemotherapy are the patients most likely to receive the greatest clinical benefit from chemotherapy treatment.

A correlation of HER-2/neu amplification/over-expression and favorable response to neo-adjuvant chemotherapy has been shown in clinical studies [17-19]. Tinari [16] explained that the predictive value of HER-2 is not direct, but rather dependent on the TopoII genes status. The value of HER-2/neu status as a predictor of response to anthracycline-based chemotherapy is still a matter of debate [20-22]. These contrasting results provide a plausible functional link between some important protein change expressions with chemotherapy response. The expression of C-erbB-2 might reflect the effect of the drug. Decreased levels of C-erbB-2 protein may result from down-regulation of transcription, increased degradation, or allele deletion, owing to the effects of anthracyclines [23]. The decreased expressions of C-erbB-2 and CXCR4 in specimens that tend to be correlated with pathological response to neo-adjuvant chemotherapy may be explained in our study, or at least in part. Not all breast cancer patients respond in the same manner to chemotherapy, and the down-regulated expressions of CXCR4 and C-erbB-2 after primary chemotherapy may be a sensitive response to treatment. The change expression of markers such as C-erbB-2 and/or CXCR4 in breast cancer may reflect the response to primary treatment with anthracyclines or other drugs such as taxanes.

A validated chemosensitivity predictive or evaluated method could be useful in deciding which treatment may or may not be effective in patients with advanced disease. There are a limited number of studies that have addressed the significance of changes in biological marker expressions as a consequence of primary chemotherapy in breast cancer.

This study suggested that decreased expressions of c-erbB-2 and CXCR4 may be a novel mechanism of chemotherapy; the changes of these objective markers may be useful in evaluating the clinical response of neo-adjuvant chemotherapy and selecting chemotherapy regimen for postoperative chemotherapy in breast cancer. Our results provide, for the first time, clear evidence that reduced or stable expressions of c-erbB2 and CXCR4 after combination chemotherapy may be an important factor to treatment. According to our results, if decreased CXCR4 and HER-2 expressions are present in a tumor, treatment could be continued, whereas absence of change or increased CXCR4 and HER-2 expressions would imply that patients should be treated with another chemotherapeutic regimen. If changes in c-erbB2 and CXCR4 expressions are confirmed as being associated with clinical survival in patients in operable breast cancer, then these may be useful objective response markers to evaluate the clinical response of neo-adjuvant chemotherapy and select the appropriate chemotherapy regimen for postoperative chemotherapy. It will be important to further evaluate this concept in future studies. Owing to the limited number of patients and the retrospective nature of this study, large, prospective studies are warranted to validate the observations from this study.

## Conclusions

As demonstrated in our study, after breast cancer patients were treated with neo-adjuvant systemic therapy, decreased expressions of C-erbB2, ER-α and CXCR4 were observed. Down-regulated expressions of c-erbB-2 and CXCR4 may be a novel mechanism of chemotherapy; the changes of these objective markers may be useful in evaluating the clinical response of neo-adjuvant chemotherapy in breast cancer.

## Competing interests

The authors have no competing interest to declare.
